# Crystal structure of 3-bromo­methyl-2-chloro-6-(di­bromo­meth­yl)quinoline

**DOI:** 10.1107/S2056989015008002

**Published:** 2015-04-30

**Authors:** Kasirajan Gayathri, Palathurai S. Mohan, Judith A. K. Howard, Hazel A. Sparkes

**Affiliations:** a Department of Chemistry, Bharathiar University, Coimbatore 641 046, Tamil Nadu, India; b Department of Chemistry, University of Durham, South Road, Durham DH1 3LE, England; c Univeristy of Bristol, Department of Chemistry, Cantock’s Close, Bristol BS8 1TS, England

**Keywords:** crystal structure, quinoline, bromo­quinolines, halogen–halogen contacts, Br⋯Cl contacts, Br⋯N contacts, C—H⋯Br hydrogen bonds, π–π inter­actions

## Abstract

In the title compound, C_11_H_7_Br_3_ClN, the quinoline ring system is approximately planar (r.m.s. = 0.011 Å). In the crystal, mol­ecules are linked by C—H⋯Br inter­actions forming chains along [10-1]. The chains are linked by C—H⋯π and π–π inter­actions involving inversion-related pyridine rings [inter­centroid distance = 3.608 (4) Å], forming sheets parallel to (10-1). Within the sheets, there are two significant short inter­actions involving a Br⋯Cl contact of 3.4904 (18) Å and a Br⋯N contact of 3.187 (6) Å, both of which are significantly shorter than the sum of their van der Waals radii.

## Related literature   

The title compound is an important inter­mediate in the manufacture of materials such as organic light-emitting devices. For the synthesis of the title compound, see: Jones (1977 ▸); Lyle *et al.* (1972 ▸). For the biological activity of quinoline derivatives, see: Chauhan & Srivastava (2001 ▸); Ferrarini *et al.* (2000 ▸); Chen *et al.* (2001 ▸); Sahin *et al.* (2008 ▸).
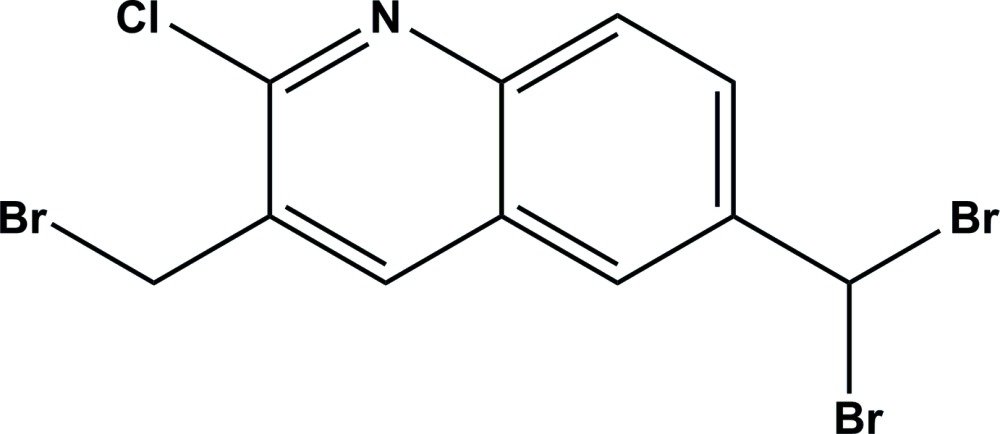


## Experimental   

### Crystal data  

C_11_H_7_Br_3_ClN
*M* *_r_* = 428.36
Monoclinic, 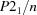

*a* = 8.9042 (5) Å
*b* = 9.3375 (4) Å
*c* = 15.5107 (7) Å
β = 104.553 (5)°
*V* = 1248.23 (11) Å^3^
*Z* = 4
Mo *K*α radiation
μ = 9.88 mm^−1^
*T* = 120 K
0.42 × 0.36 × 0.30 mm

### Data collection  

Oxford Diffraction Xcalibur Sapphire3 Gemini ultra diffractometer
Absorption correction: analytical (*CrysAlis PRO*; Oxford Diffraction, 2010 ▸) *T*_min_ = 0.056, *T*_max_ = 0.153
8597 measured reflections
2259 independent reflections
1889 reflections with *I* > 2σ(*I*)
*R*_int_ = 0.029

### Refinement  

*R*[*F* ^2^ > 2σ(*F* ^2^)] = 0.051
*wR*(*F* ^2^) = 0.143
*S* = 1.09
2259 reflections
145 parameters
H-atom parameters constrained
Δρ_max_ = 1.65 e Å^−3^
Δρ_min_ = −1.19 e Å^−3^

### 

Data collection: *CrysAlis PRO* (Oxford Diffraction, 2010 ▸); cell refinement: *CrysAlis PRO*; data reduction: *CrysAlis PRO*; program(s) used to solve structure: *SHELXS97* (Sheldrick, 2008 ▸); program(s) used to refine structure: *SHELXL2014* (Sheldrick, 2015 ▸); molecular graphics: *PLATON* (Spek, 2009 ▸) and *Mercury* (Macrae *et al.*, 2008 ▸); software used to prepare material for publication: *SHELXL2014* and *PLATON*.

## Supplementary Material

Crystal structure: contains datablock(s) I, Global. DOI: 10.1107/S2056989015008002/su5096sup1.cif

Structure factors: contains datablock(s) I. DOI: 10.1107/S2056989015008002/su5096Isup2.hkl

Supporting information file. DOI: 10.1107/S2056989015008002/su5096Isup3.cml

**Fig. 1.**
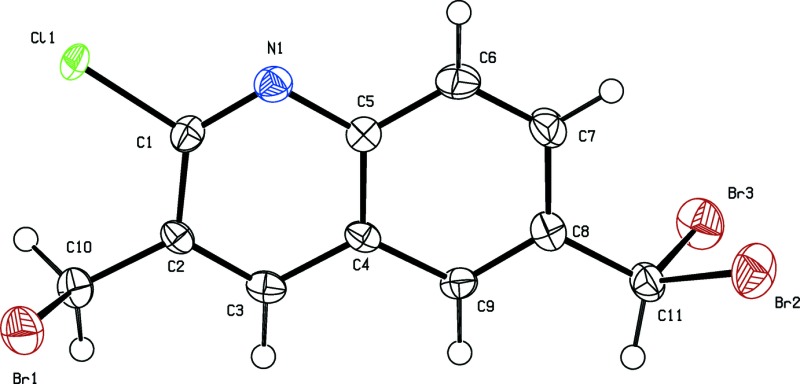
The molecular structure of the title compound, with atom labelling. Displacement ellipsoids are drawn at the 50% probability level.

**Fig. 2.**
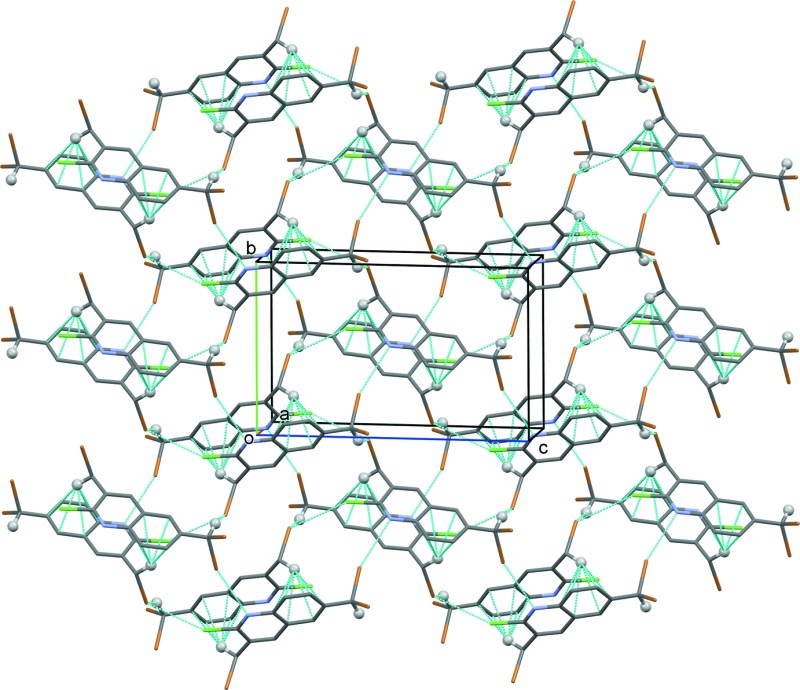
A view along the a axis of the crystal packing of the title compound. The C—H···Br hydrogen bonds, C—H···π interactions (Table 1) and the Br···Cl and Br···N short contacts are shown as dashed lines.

. DOI: 10.1107/S2056989015008002/su5096fig1.tif
The mol­ecular structure of the title compound, with atom labelling. Displacement ellipsoids are drawn at the 50% probability level.

a . DOI: 10.1107/S2056989015008002/su5096fig2.tif
A view along the *a* axis of the crystal packing of the title compound. The C—H⋯Br hydrogen bonds, C—H⋯π inter­actions (Table 1) and the Br⋯Cl and Br⋯N short contacts are shown as dashed lines.

CCDC reference: 902598

Additional supporting information:  crystallographic information; 3D view; checkCIF report

## Figures and Tables

**Table 1:** Hydrogen-bond geometry (, ) *Cg*2 is the centroid of the C4C9 ring.

*D*H*A*	*D*H	H*A*	*D* *A*	*D*H*A*
C11H11Br1^i^	1.00	2.92	3.709(8)	137
C10H10*B* *Cg*2^ii^	0.99	2.70	3.438(8)	131

Symmetry codes: (i) 
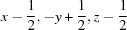
; (ii) 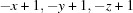.

## supplementary crystallographic information

### S1. Synthesis and crystallization

The title compound was prepared in line with literature methods (Jones, 1977; Lyle *et al.*, 1972). 2-Chloro-3,6-di­methyl-quinoline (0.001 mole) was dissolved in CCl_4_. To this benzoyl peroxide (50 mg) was added and the mixture was stirred under ice-cold conditions. To this mixture *N*-bromo­succinimide (0.005 mole) was added portion wise. The whole mixture was further stirred under ice-cold condition for 1 h. The reaction mixture was then refluxed for about 10 hours. After completion of the reaction, the succinimide was removed (it was insoluble in CCl_4_) by filtration and washed with 20 ml of CCl_4_. The contents of the filtrate were reduced to half, and the residue was chromatographed on silica gel using petroleum ether and ethyl acetate as eluent (99:1), which gave the titled product (yield: 52%). The white solid obtained was then recrystallized using acetone yielding colourless block-like crystals.

### S2. Refinement

Crystal data, data collection and structure refinement details are summarized in Table 1. H atoms were included in calculated positions and refined using a riding model: C—H = 0.95 - 1.0 Å with *U_iso_*(H) = 1.2*U_eq_*(C). The highest peak in the final difference Fourier map (1.654 eÅ^-3^) is close to atom Cl1. Attempts to split this atom were unsuccessful.

### S3. Structural commentary

The presence of the quinoline skeleton in the frameworks of pharmacologically active compounds and natural products has spurred on the development of different strategies for their synthesis (Chauhan *et al.*, 2001; Ferrarini *et al.*, 2000; Chen *et al.*, 2001). Bromo­quinolines have been of inter­est for chemists as precursors for heterocyclic compounds with multifunctionality, giving accessibility to a wide variety of compounds. These building blocks have been used in medicinal chemistry as starting materials for numerous compounds with pharmacological activity (Sahin *et al.*, 2008).

The molecular structure of the title compound is shown in Fig. 1. The quinoline ring is planar (r.m.s. = 0.011 Å).

In the crystal, molecules are linked by C—H···Br inter­actions forming chains along [101]; Table 1 and Fig. 2. The chains are linked by C—H···π (Table 1), and π-π inter­actions involving inversion related pyridine rings (N1/C1—C5) with an inter-centroid distance of 3.608 (4) Å, forming sheets parallel to (101). Within the sheets, there are two significant short inter­actions: A Br1···Cl^i^ contact of 3.4904 (18) Å and a Br3···N^ii^ contact of 3.187 (6) Å [symmetry codes: (i) -x+3/2, y-1/2, -z+3/2; (ii) x-1/2, -y+3/2, z-1/2], both are significantly shorter than the sum of their van der Waals radii [1.85 Å for Br; 1.75 Å for Cl; 1.55 Å for N; PLATON (Spek, 2009)].

### Crystal data

**Table:**

C_11_H_7_Br_3_ClN	*F*(000) = 808
*M**_r_* = 428.36	*D*_x_ = 2.279 Mg m^−^^3^
Monoclinic, *P*2_1_/*n*	Mo *K*α radiation, λ = 0.71073 Å
*a* = 8.9042 (5) Å	Cell parameters from 4382 reflections
*b* = 9.3375 (4) Å	θ = 2.6–29.0°
*c* = 15.5107 (7) Å	µ = 9.88 mm^−^^1^
β = 104.553 (5)°	*T* = 120 K
*V* = 1248.23 (11) Å^3^	Block, colourless
*Z* = 4	0.42 × 0.36 × 0.30 mm

### Data collection

**Table:**

Oxford Diffraction Xcalibur Sapphire3 Gemini ultra diffractometer	2259 independent reflections
Radiation source: Enhance (Mo) X-ray Source	1889 reflections with *I* > 2σ(*I*)
Graphite monochromator	*R*_int_ = 0.029
Detector resolution: 16.1511 pixels mm^-1^	θ_max_ = 25.3°, θ_min_ = 2.4°
ω scans	*h* = −10→10
Absorption correction: analytical (*CrysAlis PRO*; Oxford Diffraction, 2010)	*k* = −11→11
*T*_min_ = 0.056, *T*_max_ = 0.153	*l* = −18→12
8597 measured reflections	

### Refinement

**Table:**

Refinement on *F*^2^	Primary atom site location: structure-invariant direct methods
Least-squares matrix: full	Secondary atom site location: difference Fourier map
*R*[*F*^2^ > 2σ(*F*^2^)] = 0.051	Hydrogen site location: inferred from neighbouring sites
*wR*(*F*^2^) = 0.143	H-atom parameters constrained
*S* = 1.09	*w* = 1/[σ^2^(*F*_o_^2^) + (0.0713*P*)^2^ + 10.6242*P*] where *P* = (*F*_o_^2^ + 2*F*_c_^2^)/3
2259 reflections	(Δ/σ)_max_ < 0.001
145 parameters	Δρ_max_ = 1.65 e Å^−^^3^
0 restraints	Δρ_min_ = −1.19 e Å^−^^3^

### Special details

**Table:**

Geometry. All esds (except the esd in the dihedral angle between two l.s. planes) are estimated using the full covariance matrix. The cell esds are taken into account individually in the estimation of esds in distances, angles and torsion angles; correlations between esds in cell parameters are only used when they are defined by crystal symmetry. An approximate (isotropic) treatment of cell esds is used for estimating esds involving l.s. planes.

### Fractional atomic coordinates and isotropic or equivalent isotropic displacement parameters (Å^2^)

**Table:**

	*x*	*y*	*z*	*U*_iso_*/*U*_eq_	
Br1	0.63474 (10)	0.04489 (9)	0.60991 (6)	0.0370 (3)	
Br3	0.45280 (10)	0.74291 (9)	0.12751 (6)	0.0381 (3)	
Br2	0.70035 (11)	0.53886 (11)	0.08299 (6)	0.0429 (3)	
Cl1	0.8069 (2)	0.38929 (19)	0.67965 (10)	0.0226 (4)	
N1	0.7951 (7)	0.4817 (7)	0.5207 (4)	0.0242 (13)	
C1	0.7245 (8)	0.3973 (8)	0.5630 (4)	0.0215 (15)	
C2	0.5903 (8)	0.3156 (7)	0.5249 (4)	0.0179 (14)	
C3	0.5309 (8)	0.3292 (7)	0.4361 (5)	0.0201 (14)	
H3	0.4411	0.2763	0.4074	0.024*	
C4	0.6012 (8)	0.4213 (7)	0.3857 (4)	0.0175 (14)	
C5	0.7359 (8)	0.4957 (8)	0.4305 (5)	0.0197 (14)	
C6	0.8084 (8)	0.5892 (9)	0.3828 (5)	0.0274 (17)	
H6	0.8991	0.6397	0.4129	0.033*	
C7	0.7505 (9)	0.6079 (9)	0.2946 (5)	0.0279 (17)	
H7	0.8002	0.6722	0.2631	0.034*	
C8	0.6160 (8)	0.5326 (8)	0.2483 (5)	0.0217 (15)	
C9	0.5450 (8)	0.4423 (7)	0.2927 (4)	0.0201 (14)	
H9	0.4555	0.3916	0.2612	0.024*	
C10	0.5174 (9)	0.2212 (8)	0.5792 (5)	0.0234 (15)	
H10A	0.4102	0.1981	0.5456	0.028*	
H10B	0.5120	0.2718	0.6344	0.028*	
C11	0.5499 (9)	0.5552 (8)	0.1511 (5)	0.0250 (16)	
H11	0.4683	0.4809	0.1295	0.030*	

### Atomic displacement parameters (Å^2^)

**Table:**

	*U*^11^	*U*^22^	*U*^33^	*U*^12^	*U*^13^	*U*^23^
Br1	0.0423 (5)	0.0311 (5)	0.0363 (5)	0.0018 (4)	0.0073 (4)	0.0085 (3)
Br3	0.0415 (5)	0.0292 (5)	0.0416 (5)	0.0098 (4)	0.0068 (4)	0.0091 (3)
Br2	0.0437 (5)	0.0546 (6)	0.0369 (5)	0.0031 (4)	0.0225 (4)	−0.0033 (4)
Cl1	0.0252 (9)	0.0296 (9)	0.0114 (7)	−0.0033 (7)	0.0013 (6)	0.0017 (6)
N1	0.022 (3)	0.025 (3)	0.024 (3)	−0.001 (3)	0.003 (2)	0.000 (3)
C1	0.020 (4)	0.026 (4)	0.018 (3)	0.001 (3)	0.005 (3)	−0.001 (3)
C2	0.019 (3)	0.012 (3)	0.023 (3)	0.002 (3)	0.007 (3)	0.002 (3)
C3	0.020 (3)	0.012 (3)	0.027 (4)	0.003 (3)	0.004 (3)	−0.001 (3)
C4	0.017 (3)	0.013 (3)	0.024 (3)	0.001 (3)	0.008 (3)	−0.002 (3)
C5	0.019 (3)	0.020 (3)	0.021 (3)	0.002 (3)	0.005 (3)	0.000 (3)
C6	0.018 (4)	0.030 (4)	0.031 (4)	−0.003 (3)	0.000 (3)	−0.002 (3)
C7	0.023 (4)	0.033 (4)	0.029 (4)	−0.007 (3)	0.008 (3)	0.004 (3)
C8	0.022 (4)	0.018 (4)	0.026 (4)	0.005 (3)	0.010 (3)	0.002 (3)
C9	0.021 (4)	0.016 (3)	0.020 (3)	0.001 (3)	−0.001 (3)	0.000 (3)
C10	0.024 (4)	0.023 (4)	0.025 (4)	0.003 (3)	0.008 (3)	0.003 (3)
C11	0.030 (4)	0.021 (4)	0.026 (4)	−0.001 (3)	0.009 (3)	0.004 (3)

### Geometric parameters (Å, º)

**Table:**

Br1—C10	1.944 (7)	C4—C9	1.417 (9)
Br3—C11	1.948 (7)	C5—C6	1.404 (11)
Br2—C11	1.910 (8)	C6—C7	1.345 (10)
Cl1—C1	1.776 (7)	C6—H6	0.9500
N1—C1	1.286 (10)	C7—C8	1.419 (10)
N1—C5	1.371 (9)	C7—H7	0.9500
C1—C2	1.416 (10)	C8—C9	1.343 (10)
C2—C3	1.352 (10)	C8—C11	1.489 (10)
C2—C10	1.478 (10)	C9—H9	0.9500
C3—C4	1.409 (10)	C10—H10A	0.9900
C3—H3	0.9500	C10—H10B	0.9900
C4—C5	1.409 (10)	C11—H11	1.0000
			
C1—N1—C5	117.8 (6)	C6—C7—H7	119.7
N1—C1—C2	126.0 (6)	C8—C7—H7	119.7
N1—C1—Cl1	114.6 (5)	C9—C8—C7	119.9 (7)
C2—C1—Cl1	119.5 (5)	C9—C8—C11	119.4 (7)
C3—C2—C1	116.6 (6)	C7—C8—C11	120.7 (7)
C3—C2—C10	121.5 (6)	C8—C9—C4	121.2 (6)
C1—C2—C10	121.9 (6)	C8—C9—H9	119.4
C2—C3—C4	120.5 (6)	C4—C9—H9	119.4
C2—C3—H3	119.8	C2—C10—Br1	111.0 (5)
C4—C3—H3	119.8	C2—C10—H10A	109.4
C5—C4—C3	118.0 (6)	Br1—C10—H10A	109.4
C5—C4—C9	118.2 (6)	C2—C10—H10B	109.4
C3—C4—C9	123.8 (6)	Br1—C10—H10B	109.4
N1—C5—C6	119.2 (6)	H10A—C10—H10B	108.0
N1—C5—C4	121.2 (6)	C8—C11—Br2	113.3 (5)
C6—C5—C4	119.6 (6)	C8—C11—Br3	111.2 (5)
C7—C6—C5	120.5 (7)	Br2—C11—Br3	107.9 (3)
C7—C6—H6	119.8	C8—C11—H11	108.1
C5—C6—H6	119.8	Br2—C11—H11	108.1
C6—C7—C8	120.6 (7)	Br3—C11—H11	108.1
			
C5—N1—C1—C2	0.4 (11)	N1—C5—C6—C7	−178.3 (7)
C5—N1—C1—Cl1	−178.6 (5)	C4—C5—C6—C7	−0.2 (11)
N1—C1—C2—C3	−0.7 (11)	C5—C6—C7—C8	−0.5 (12)
Cl1—C1—C2—C3	178.3 (5)	C6—C7—C8—C9	0.4 (12)
N1—C1—C2—C10	180.0 (7)	C6—C7—C8—C11	178.8 (7)
Cl1—C1—C2—C10	−1.0 (9)	C7—C8—C9—C4	0.5 (11)
C1—C2—C3—C4	−0.2 (10)	C11—C8—C9—C4	−177.9 (6)
C10—C2—C3—C4	179.1 (6)	C5—C4—C9—C8	−1.2 (10)
C2—C3—C4—C5	1.2 (10)	C3—C4—C9—C8	179.4 (7)
C2—C3—C4—C9	−179.4 (7)	C3—C2—C10—Br1	103.9 (7)
C1—N1—C5—C6	178.7 (7)	C1—C2—C10—Br1	−76.8 (8)
C1—N1—C5—C4	0.7 (10)	C9—C8—C11—Br2	−131.1 (6)
C3—C4—C5—N1	−1.5 (10)	C7—C8—C11—Br2	50.5 (8)
C9—C4—C5—N1	179.1 (6)	C9—C8—C11—Br3	107.2 (7)
C3—C4—C5—C6	−179.5 (7)	C7—C8—C11—Br3	−71.3 (8)
C9—C4—C5—C6	1.0 (10)		

### Hydrogen-bond geometry (Å, º)

Cg2 is the centroid of the C4–C9 ring.

**Table:**

*D*—H···*A*	*D*—H	H···*A*	*D*···*A*	*D*—H···*A*
C11—H11···Br1^i^	1.00	2.92	3.709 (8)	137
C10—H10*B*···*Cg*2^ii^	0.99	2.70	3.438 (8)	131

Symmetry codes: (i) *x*−1/2, −*y*+1/2, *z*−1/2; (ii) −*x*+1, −*y*+1, −*z*+1.
