# Supplementary figures and images for: Crystal structure of 3-bromo­methyl-2-chloro-6-(di­bromo­meth­yl)quinoline
Source: Acta Crystallogr E Crystallogr Commun. 2015 Apr 30;71(Pt 5):o354–5. doi: 10.1107/S2056989015008002 (PMC4420095; doi:10.1107/S2056989015008002)

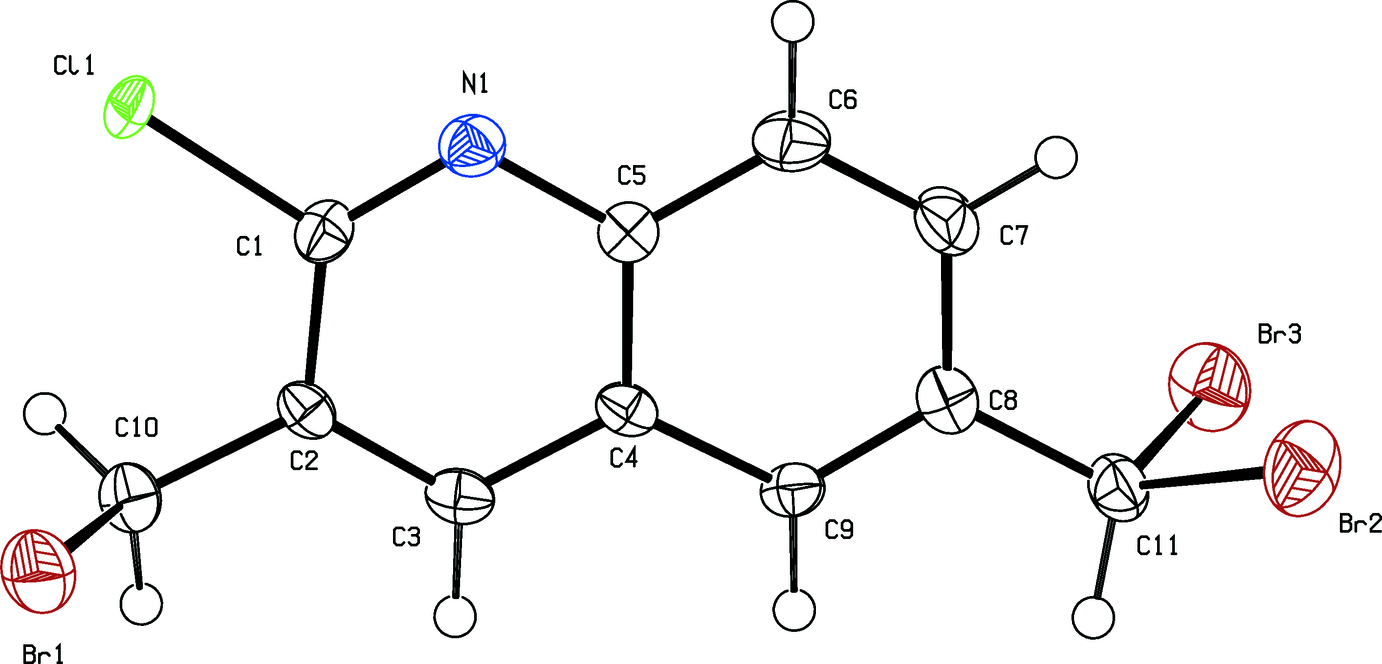

Supplement: Supplementary file 4 [file e-71-0o354-fig1.tif]

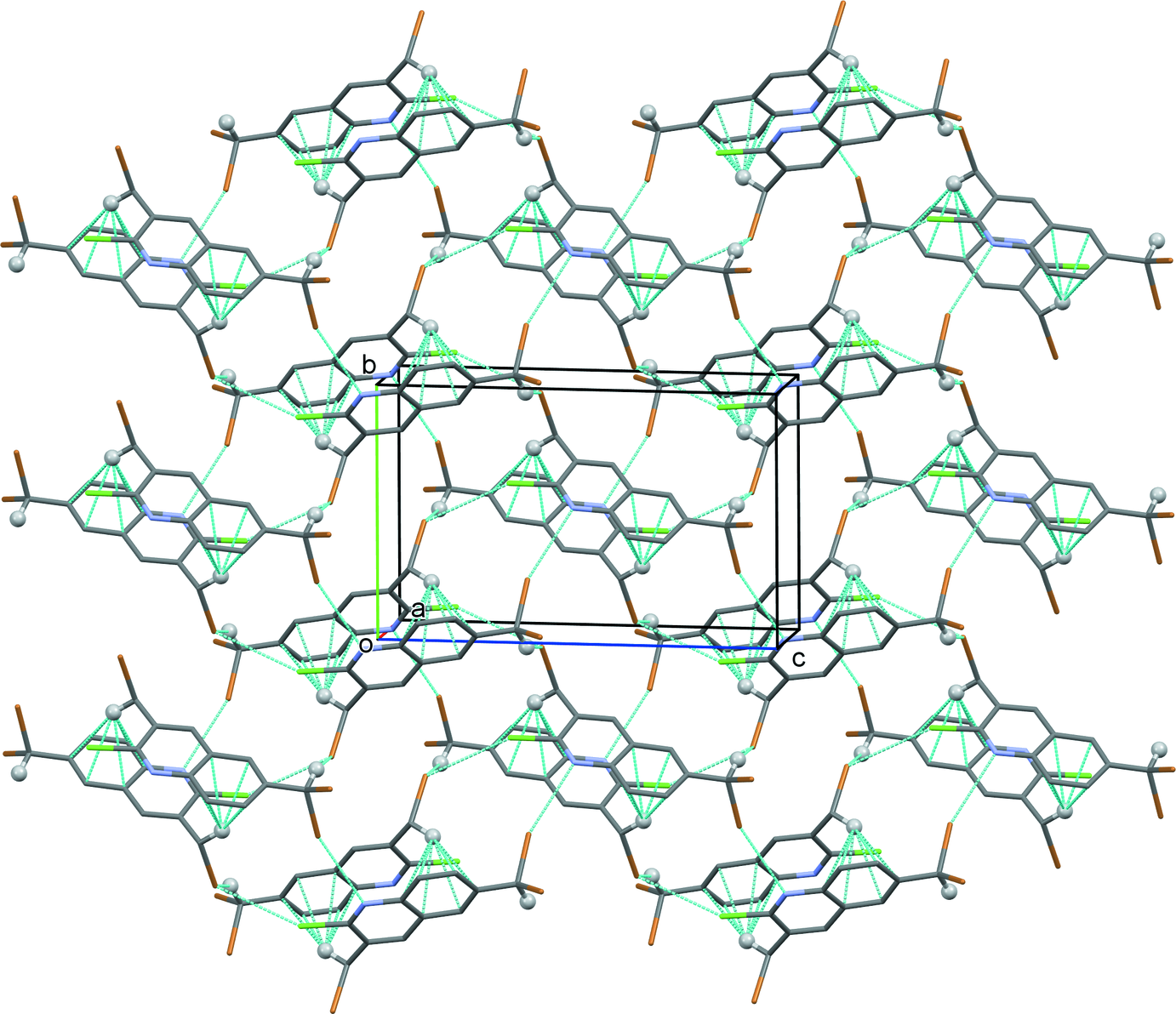

Supplement: Supplementary file 5 [file e-71-0o354-fig2.tif]
